# I am *ready* to see you now, Doctor! A mixed‐method study of the Let's Discuss Health website implementation in Primary Care

**DOI:** 10.1111/hex.13158

**Published:** 2020-12-07

**Authors:** Marie‐Thérèse Lussier, Claude Richard, Fatoumata Binta Diallo, Nathalie Boivin, Catherine Hudon, Élie Boustani, Holly Witteman, Jalila Jbilou

**Affiliations:** ^1^ Department of Family Medicine and Emergency Medicine University of Montreal Montreal QC Canada; ^2^ Laval Integrated Health and Social Services Centre (Centre de santé et des services sociaux de Laval) Groupe de recherche sur les transformations des pratiques cliniques et organisationnelles Laval QC Canada; ^3^ École réseau de Science infirmière (ÉRSI) University of Moncton Moncton NB Canada; ^4^ Department of Family Medicine and Emergency Medicine University of Sherbrooke Sherbrooke QC Canada; ^5^ Department of Family Medicine and Emergency Medicine Laval University Laval QC Canada; ^6^ Centre de formation médicale du Nouveau Brunswick, Université de Sherbrooke École de psychologie, University of Moncton Moncton NB Canada

**Keywords:** doctor‐patient relationship, medical encounter, patient activation, patient engagement, patient participation, personal health records, web strategies

## Abstract

**Background:**

*Let's Discuss Health* (LDH) is a website that encourages patients to prepare their health‐care encounters by providing communication training, review of topics and questions that are important to them.

**Objective:**

To describe LDH implementation during primary care (PC) visits for chronic illnesses.

**Methods:**

Design: Descriptive mixed‐method study. Setting: 6 PC clinics. Participants: 156 patients and 51 health‐care providers (HCP). Intervention: LDH website implementation. Outcome Measures: Perceived quality and usefulness of LDH; perceived quality of HCP‐patient communication; patient activation; LDH integration in routine PC practices and barriers to its use.

**Results:**

Patients reported a positive perception of the website in that it helped them to adopt an active role in the encounters; recall their visit agenda and reduce encounter‐related stress; feel more confident to ask questions, feel more motivated to prepare their future medical visits and improve their chronic illness management. However, a certain disconnect emerged between HCP and patient perceptions as to the value of LDH in promoting a sense of partnership and collaboration. The main barriers to the use of LDH are HCP lack of interest, limited access to technology, lack of time and language barriers.

**Conclusion:**

Our findings indicate that it is advantageous for patients to prepare their medical encounters. However, the study needs to be replicated in other medical environments using larger and more diverse samples.

**Patient and Public Contribution:**

Patient partners were involved in the conduct of this study.

## 1 BACKGROUND

High‐quality communication between patients and health providers is essential for better management of chronic illness. In Canada, as in many other countries, much of such care occurs in primary care (PC). It is estimated that 40% of health‐care visits in PC include a follow‐up for chronic health problems.^1^ The prevalence of multimorbidity in this context is high.^2^, ^3^, ^4^ Both of these characteristics undeniably increase the complexity of encounters. However, the average duration of a health‐care visit in this context is short, approximately 15 minutes,^5^ and the majority of patients do not know how medical appointments are structured and so have difficulty preparing for them.^5^, ^6^, ^7^, ^8^, ^9^ Helping patients prepare for their PC health‐care encounters thus provides an interesting avenue to improve the effectiveness of chronic illness health‐care visits.

Indeed, the quality of communication between patients with chronic illnesses and their health‐care providers (HCP) is recognized as playing an important role in the management of their illness.^10^, ^11^, ^12^, ^13^, ^14^, ^15^ Yet, compared with epidemiological studies, relatively few studies have specifically focused on the interactions between patients and HCP during their encounters.^15^ Data from research on communication reveal how important it is for the HCP to play the role of partner or facilitator^16^ in order to arrive at a mutual understanding of the nature of the illness and to engage in a shared decision process on the best course of treatment.^17^, ^18^, ^19^, ^20^ In recent years, the idea of focusing directly on patients’ communication skills instead of those of HCP to improve the quality of the encounter has emerged.^7^, ^21^ Patient *activation* (i.e. encouraging patients to take an active role in the management of their chronic illness) is one of the best ways to reach this goal.^21^, ^22^, ^23^, ^24^, ^25^ The evidence indicates that patient *activation* improves participation during health‐care encounters, recall of the information discussed, treatment adherence and other health‐care outcomes.^7^, ^15^, ^21^, ^22^, ^26^, ^27^ Thus, activation can increase patient commitment to the self‐management of their chronic illnesses.^23^, ^24^, ^28^ However, the majority of studies on patient activation have required the intervention of a trainer before the patient's planned HCP appointment. This approach is complicated to organize, imposes a rigid schedule on the patient and can lead to considerable costs, all significant challenges to its adoption. Online interventions present a potential solution to overcoming these barriers.^29^

*Let's Discuss Health* (LDH) is a French Canadian website that encourages patients to take an active role in managing their health and supports their collaboration with their caregivers. It is akin to a ‘stand‐alone’ electronic personal health record (PHR) with which it shares the aim of allowing patients to hold their own health record including active diagnoses or problem list, medication list and personal and family medical history. A major distinction between PHR and LDH is that it is not, as yet, connected to the HCPs’ electronic medical record (EMR). LDH does not allow information transfer from the EMR to the PHR nor does it provide an electronic communication channel between patients and providers in between clinic visits as in many patient portals.^30^ However, it fills an important gap not covered in almost all PHR, including the Carnet santé Québec^31^ in that it guides patients in the preparation of their visit agenda for time‐constrained medical visits and in setting their priorities.^32^ LDH encourages patients to become active participants in their health‐care encounters by providing communication skills coaching as well as ‘walking them’ through the encounter process. The LDH website includes three distinct modules: My PACE skills (*P*repare, *A*sk questions, *C*heck understanding, *E*xpress concerns), My Appointments and My Health Booklet. The PACE module describes and demonstrates useful communication strategies that can improve patient participation in the encounter. My Appointments module assists the patient in preparing his/her medical consultation by prompting him/her to identify and, if there is more than one reason, to prioritize the reasons he/she is seeking a consultation for. For each reason, the system encourages the patient to provide context and details. Moreover, the system requests that the patient list all medications used and questions he/she wants to ask the HCP. My Health Booklet is a module in which the patient may insert information regarding his/her past medical history, vaccinations, results of specialist consultations, etc This module is particularly useful if the encounter for which the patient is preparing is an encounter with a new HCP. Once the patient has completed My Appointment module, the system then generates a ‘visit preparation summary’ that can be visualized on his/her mobile device or printed. This visit summary can either be consulted by the patient during the visit or shared by him/her with the HCP. The development and validation of the site are based on the best practices in the field.^33^, ^34^, ^35^ Content development is based on recognized health communication models.^16^, ^20^, ^36^ Patients with chronic illnesses and their HCP were consulted at every crucial step of LDH’s development process and are described elsewhere.^37^ Even though LDH was judged extremely useful and pertinent by a group of test users during a validation study,^37^ this does not guarantee it will be adopted into PC clinical practice. The potential for integration of this tool in regular medical visits for patients with chronic illnesses needs to be assessed.

The goal of this study is to describe the implementation of LDH in PC clinics and its adoption during routine family medicine follow‐up visits of patients with chronic illnesses. More specifically, it aims to: (a) describe patient experience of LDH website use; (b) identify, from both patient and HCP perspectives, the association between patient preparation with LDH and patient activation, as well as with the unfolding of the visit with the HCP; and (c) identify, from patients, HCP and clinic project coordinators’ perspectives**,** the facilitators and barriers to the use of LDH tools in this clinical context.

## 2 METHODS

### 2.1 Conceptual frameworks

This study rested on two theoretical frameworks. The Reach, Effectiveness, Adoption, Implementation, and Maintenance framework (RE‐AIM)^38^, ^39^, ^40^ refers to the degree to which the intervention is integrated into current practices and established organizational policies, whereas the development of training tools for HCP and the conditions favourable to their implementation in clinics was based on the implementation of the organizational change model developed by Rondeau et al (2008).^41^ This model presents the issues associated with change according to the organized action perspective. Thus, Rondeau's model was used in conjunction with the RE‐AIM framework for the study of the implementation and adoption of the LDH.

### 2.2 Study design and participants

This was a mixed‐method descriptive study to explore the use of LDH by PC patients with chronic illnesses. We enrolled 6 PC clinics who had demonstrated an interest in integrating LDH into their routine practices. These clinics were located in Québec (n = 3) and New Brunswick (n = 3), two provinces in Canada with French‐speaking populations. Participating clinics varied in terms of geographic area (urban, semi‐urban, rural), organizational structures, team and practice size and characteristics of the population served.

Most participating clinics, either academic or community‐based, involved interdisciplinary teams, namely practicing family physicians, family medicine residents and nurses involved in the management of these patients. The invitation to participate in the study was thus sent to all these types of HCP. Anyone who was absent for a **6‐ to 12‐month** period during the implementation and data collection phases were excluded from the study. All six clinics identified a ‘project coordinator’, a well‐established clinic secretary or nurse who was to be the local contact person during the course of the project. All patients aged 18 years or older, with at least one chronic illness and a scheduled follow‐up appointment with a participating HCP during the study period (June 2015 to September 2016), were invited to participate. Other inclusion criteria were understanding, speaking and reading French and having basic Internet skills or having access to someone who did. Patients considered incapable of giving informed consent were excluded from the study.

### 2.3 Study conduct

**Figure 1:**
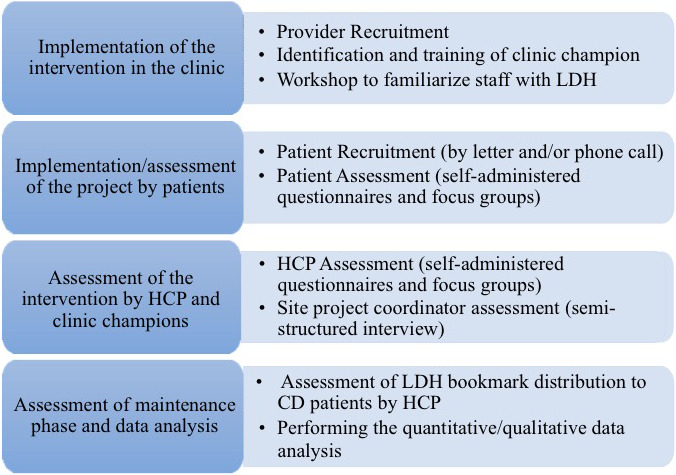
Study phase description

The study was conducted in four phases as described in Figure 1. Here, we are reporting on the first three phases. First, a clinic ‘project coordinator’ (secretary or nurse) was trained in the following tasks: (a) invite patients to participate in the study; (b) motivate them to prepare their scheduled encounters with LDH; and (c) to support HCP. This was necessary because of the limited resources available for this project. The project coordinator, in certain sites, could share these responsibilities during the recruitment phase with either a trained medical student or family medicine resident. A 1‐hour interactive workshop was delivered in order to familiarize HCP with LDH. The workshop included: (a) a presenttaion of the website and available promotional materials (bookmarks and posters); (b) a discussion of the ‘activated’ patient's communication skills; (c) viewing a video of an interaction between a HCP and a patient who comes to the visit with his/her visit preparation summary (summary sheet); and (d) role‐playing scenarios to apply the communication skills observed in the video.

Patients were invited to participate in the study by clinic staff either by letter, phone or both. **The** invitation was adapted to the two models for clinic appointment setting that were *in vogue* at the time of the study. First, in the clinics with a traditional access model (where the HCP’s schedule is completely booked in advance), we mailed a personalized letter signed by the attending HCP to eligible patients who had a scheduled follow‐up appointment in the following 4 weeks. Two weeks later, a call was made by the project coordinator to check whether the patient received the letter and briefly discuss the advantages of preparing for an appointment. Second, in the clinics with Advanced Access (appointments schedule to be available in a timely fashion), the project coordinator called eligible patients to invite them to participate in the study, outline the nature of the engagement if they chose to participate and discussed the advantages of preparing their scheduled encounter. Participating patients were reminded to bring their ‘visit preparation summary’ to the visit. Patients signed their study consent form when they arrived at the clinic. In either model, the clinic's secretary called the study patients to confirm the upcoming appointment, remind them of their participation in the study and to bring their visit preparation summary. On arrival at the clinic, patients were referred to the project coordinator or the research assistant for all tasks related to data collection and study follow‐up.

### 2.4 Measures and data collection

**Figure 2:**
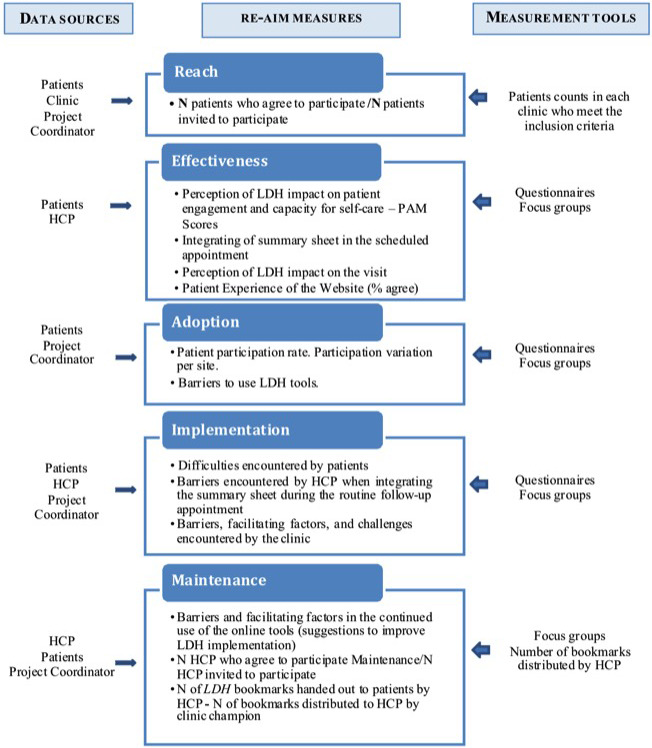
Applying the RE‐AIM model to the study

Figure 2 presents data sources and measurement tools according to data collected in keeping with the RE‐AIM framework.

*Quantitative data* were collected from patients, HCP and project coordinators.

Patients were asked to complete two short self‐administered questionnaires. The first, developed specifically for this study, asked for basic sociodemographic and clinical data, their impressions of the LDH website and its perceived usefulness for preparing their medical visit. The second questionnaire, the Patient Activation Measure (PAM 13),^28^ is a validated instrument that assesses patient self‐reported knowledge, skills and confidence in regard to the self‐management of their health or chronic condition. It consists of 13 statements with the following answer categories: Disagree strongly, Disagree, Agree, Agree strongly or Does not apply. It was administered to patients after they visited the website and prepared for their appointments. PAM scores were calculated according to guidelines provided by the PAM survey licence proprietor Insigna Health Inc.^42^ PAM scores range from 0 to 100, with higher scores indicating greater activation. Scores are categorized into four levels. Level 1 patients (score ≤ 47) are disengaged and overwhelmed; Level 2 patients (score 47.1‐55.1) lack confidence and struggle to take action; Level 3 patients (score 55.2‐66.9) begin to take action; and Level 4 patients (score ≥ 67) are maintaining healthy behaviours. Furthermore, project coordinators kept a study logbook that allowed us to document recruitment and provided information about patient participation rates. HCP filled the first self‐administered questionnaire on their sociodemographic and professional characteristics. Immediately after the encounter with a study patient, they also completed a self‐administered questionnaire to gather their impressions of the unfolding of the encounter, the value of the LDH summary sheet and its impact on the visit. This questionnaire was developed for the present study based on a similar questionnaire used in the Talking Health Together study,^15^, ^43^ a randomized trial evaluating the impact of a patient communication intervention delivered in two formats (e‐learning or e‐learning followed by a workshop) on patient participation in PC encounters, patient recall and patient health outcomes.

Project coordinators provided bookmarks, a form of LDH promotional material, to the HCP to distribute to eligible patients. We are not reporting on this phase of the study.

*Qualitative data* were collected from patients’ and HCP’s focus groups and semi‐structured interviews with project coordinators. The patient interview guide included questions designed to explore the user experience of the website and their experience of the follow‐up appointment using the visit preparation summary. The HCP interview guide, based on the organizational change model,^41^ included questions designed to explore their experience of consultations with prepared patients. All focus groups took place once the period of scheduled appointments for eligible patients had ended. Focus groups helped to: (a) assess the project's pertinence and usefulness and (b) identify facilitating factors and barriers to the implementation of LDH in routine clinical practices. We also conducted the semi‐structured interviews with the project coordinators to identify facilitating factors and barriers to the implementation of LDH in routine clinical practices. Audio recordings of all the focus groups and project coordinator interviews were made and subsequently transcribed verbatim.

### 2.5 Data analysis

*Quantitative data*: Descriptive statistical analyses were performed using SPSS software version 24. The descriptive data were obtained from the answers to the self‐administered patient and HCP questionnaires completed following the appointments. An isolated analysis of each of the six clinics was carried out, and the findings were then aggregated by province and for the study as a whole. Analyses were performed on all the variables. The perceived impact of the use of LDH on HCP‐patient communication during follow‐up visits (encounter duration, structure, visit agenda, question asking, etc), the level of patient activation, LDH integration in routine clinical practices and on the degree of patient activation, as well as the barriers to the use of LDH were described using percentages. Scores on the PAM were calculated by summing up the raw scores and mapping the sum onto a scale of 0‐100.

*Qualitative data*: Data coding and analysis were performed with QDA Miner software.^44^ A senior research assistant, involved in the communication analyses of all LDH‐related projects, was trained and supervised by one of the authors (CR). Initially, they both read the full focus group transcripts and assigned thematic codes associated with the evaluation of the LDH website (patient focus groups) and its implementation in routine clinical practices as well as its impact on the medical encounter (patient and HCP focus groups) as they emerged from the responses to the interview guide. The codes assigned were reviewed and discussed on a regular basis by this team of coders. In case of coding differences, a consensus was reached as to the thematic category that best applied. An individual analysis of each clinic was first carried out, and then, the six clinics (cases) were aggregated to identify facilitating factors and barriers to implementation. To ensure the best interpretation of the data, all the researchers, collaborators, partners (patients) and volunteer clinicians from participating sites were invited to review the analyses and thus play played a key role in the analysis process.

For data integration, we collated the data from quantitative and qualitative analyses in order to identify the similarities and the differences between the results.

Ethical approval was obtained from the Ethics Committee of the participating clinics in the two provinces. All participants (patients, HCP and project coordinators) gave written informed consent before taking part in the study.

## 3 RESULTS

### 3.1 Clinics and participants

**Figure 3:**
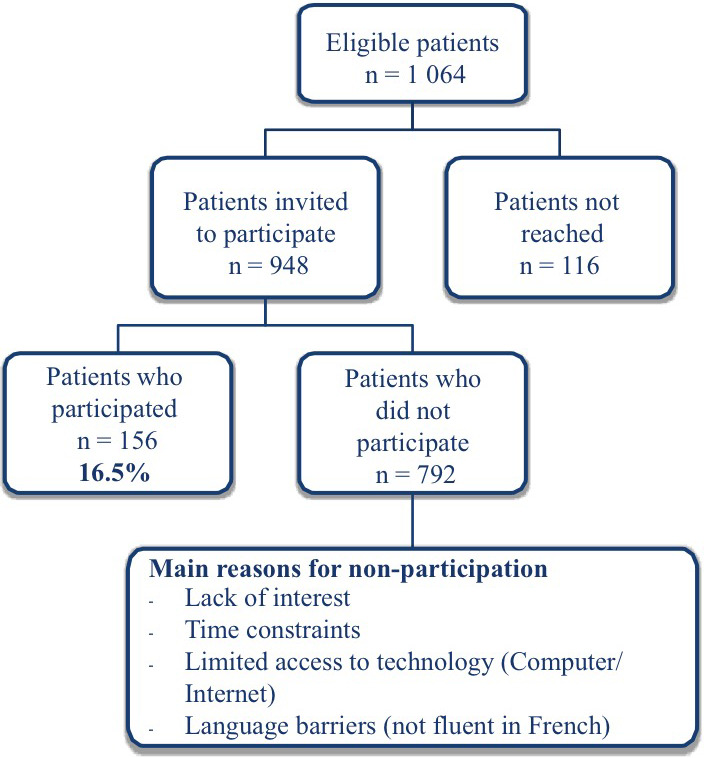
Patient recruitment flow chart

In total, 156 patients participated in the study (Figure 3), 106 were from Quebec and 50 from New Brunswick. Overall, patient participation rate was 16.5%, ranging from 7.9% to 37.3% per clinic depending on the onsite presence of dedicated research resources such as medical students or family medicine residents (data not shown). A majority of these patients were over 40 years old (84%), and 79% reported higher education levels (high school diploma). Just over half of the participants had 3 or more medical visits during the preceding year (54%).

Across all sites, 51 HCP participated in the study (range: 5 to 13 HCP per site). There was an equal representation of both novice (51%) and experienced HCP (49%). Patients and HCP sociodemographic characteristics are provided in Table 1.

**Table 1:** Sociodemographic characteristics of participants

PATIENTS (N = 156)		HCP (N = 51)	
Characteristics	N (%)	Characteristics	N (%)
Sex		Sex	
Male	69 (51)	Male	8 (19)
Female	73 (49)	Female	35 (81)
Missing	14	Age	
Age		Less than 30 y	16 (37)
40 y or less	23 (16)	31‐40 y	13 (30)
41‐60 y	68 (48)	More than 40 y	14 (33)
61‐80 y	51 (36)	‐Missing	8
Missing	14	Type of occupation	
Level of education		Physician	27 (63)
High school or less	44 (31)	Family medicine residents	13 (30)
College/Technical training	48 (44)	Nurse	3 (7)
University	49 (35)	Missing	8
Missing	15	Number of years in primary care practice	
Annual household income		5 y and less	21 (51)
Less than 40 000$	46 (35)	6‐10 y	7 (17)
40 000$‐ 79 999$	62 (48)	More than 10 y	13 (32)
More than 80 000$	22 (17)	Missing	10
Missing	26		
Duration of relationship with provider			
Less than 1 y	46 (33)		
1‐2 y	36 (25)		
3 y or more	59 (42)		
Missing	15		
Number of medical visits during the preceding year			
Less than 1	27 (19)		
2	39 (27)		
3 or more	76 (54)		
Missing	14		

By comparing the two provinces, we found that the sociodemographic characteristics of the patients were very similar with regard to age, sex and income. However, there was a slight difference in the level of education. Indeed, 29% vs 36% (high school or less) and 71% vs 64% (College/Technical training/University), in Quebec and New Brunswick, respectively.

In what follows, we report patients and HCP results, of the self‐administered questionnaires followed by the focus groups discussions. Presentation of results follows the same pattern in Tables 2, 3 and 4 in which we first report results from the patients’ self‐administered questionnaires followed by the HCP’s responses. We have organized the presentation of results following the four major themes that emerged from the data analysis: (a) impact of LDH on patient engagement; (b) value of the visit preparation summary (or summary sheet); (c) LDH user experience and its impact on the visit; and (d) identification of barriers to LDH implementation in clinics. For each theme, we show the patients and HCP perspectives expressed in the study questionnaires or during focus groups. Verbatim quotes are provided under each of the four themes. MTL translated verbatim citations from French to English.

**Table 2:** Post‐visit questionnaire data of patients’ and HCP’ perspectives of Let's Discuss Health (LDH) impact on patient engagement and capacity for self‐care

PATIENTS’ PERSPECTIVE		HCP’ PERSPECTIVE	
	% Agreement		% Agreement
I actively participated in the encounter	93%	Patient is well prepared	87%
I better manage my health conditions	91%	Patient asks relevant questions	87%
I feel motivated to prepare future visits	90%		
I intend to revisit the website	91%		

**Table 3:** Post‐visit questionnaire data of patients’ and HCP’ perspectives of the value of Let's Discuss Health (LDH) summary sheet

PATIENTS’ PERSPECTIVE		HCP’ PERSPECTIVE	
	% Agreement		% Agreement
I find the summary sheet complete	92%	Easy integration of summary sheet	80%
I find the summary sheet useful	90%	Accuracy of information on summary sheet	80%
I discussed all items on my summary sheet	99%	Incomplete content of summary sheet	33%
		Summary sheet helped to organize visit	56%
		Summary sheet provided new information that I would not have otherwise uncovered	33%

**Table 4:** Post‐visit questionnaire data of patients’ and HCP’ views about the use of Let's Discuss Health's and its impact on the visit

	PATIENTS’ PERSPECTIVE		HCP’ PERSPECTIVE	
		% Agreement		% Agreement
	I felt better understood by my provider	86%	Patients’ reason for visit are clear	87%
	I felt comfortable asking my questions	94%	Patients express their concerns clearly	89%
	I checked when I did not understand	88%		

### 3.2 Impact of using Let's Discuss Health website on patient engagement

Study patients showed a high PAM score: Level 1 (n = 4; 2.9%); Level 2 (n = 5; 3.6%); Level 3 (n = 41; 29.9%); and Level 4 (n = 87; 63.5%). Table 2 presents information on the impact of using LDH on patient engagement. According to post‐visit questionnaire data from both, patients and HCP, study patients were actively engaged in the encounter. However, opinions expressed by both groups of participants during focus group discussions differed. Patients indicated that LDH helped them play an active role in managing their health and it increased their feeling of being in a partnership with their HCP.

*‘LDH allowed me to review my medical issues and to realize the interactions and the effects that the medications I am taking have. It allowed me to note my future visits and to realize that it is important to take my health in hand’*.

*‘It (LDH) gives us the impression that it is more effective … Look, I make the effort to write it… and she (the doctor) sees it and reads it. So you say to yourself, I did my part. She saw it. She did her part. So it goes well’*.

On the other hand, many HCP did not perceive any added value to LDH use by their patients. In fact, one recurrent idea expressed by HCP was that LDH was used by patients who were probably already preparing and organizing themselves before coming in for their visit.

*‘In fact, it was not a big change … it still frequently happens that we have patients preparing for their visit’*.

*‘In any case, in my caseload of patients, I have several (patients) who prepare with lists of questions’*.

### 3.3 Perceived value of visit preparation and summary sheet

Patients agreed that the LDH visit preparation summary generated by the system was helpful to manage their medical appointment. They stated that the summary sheet was complete (92%), useful (90%) and that they discussed all items listed on it with their HCP (99%) (Table 3). During patient focus group discussions, a consensus emerged that LDH was a helpful tool to avoid oversights, reduce the stress related to a medical visit and increase patients’ sense of control as well as to help them structure their visit. Thus, findings from the patient focus groups confirmed questionnaire results.

*“With the completed listing… we don't forget. It allows you to summarize, to note and to prepare well. It allows you to have in one place the lists of drugs, the history of surgeries and hereditary problems. It works as my memory aid’*.

On the other hand, HCP questionnaire data indicated that a majority of them (80%) agreed that the sheet was easy to integrate in the flow of the medical encounter and that the information was accurate, although incomplete. It is of note that a third of HCP (33%) indicated the summary sheet helped them access information they might not have uncovered otherwise. During focus group discussions, the idea that including the summary sheet in the visit did not change their consultation routines was expressed as illustrated in the following citations.

*‘I may not necessarily have seen a difference, but it's mostly patients I have known for years’*.

Also, in terms of the impact of the LDH summary sheet on the duration of the visit, HCP views varied, some stating it shortened the visit, while others perceived it lengthened it.

*‘Didn't notice much, maybe because most of my patients I have known for a long time. Maybe it allowed them to address things they would not have addressed in a first appointment. I didn't see a difference…in consultation length’*.

A large proportion of HCP (56%) reported that the summary sheet did not help in structuring the visit. In the focus groups, they stated that their *structured* patients remained so and that their *unstructured* patients did not improve with the LDH visit preparation summary.

*‘I think patients that are more motivated regarding their health and those who perceive their health as important… I think these are the ones who are more motivated to do the extra work to prepare their appointment’*.

Some HCP said that the LDH summary sheet was no more useful than a handwritten list. However, they argued that LDH stimulated patients to list and *prioritize* the problems they wished to discuss with them. Others noted that visit preparation with LDH stimulated patient reflection that could, in turn, help clarify the information they sought.

*‘I have several patients who prepare lists of questions. It is not as elaborate as the preparation made there (with LDH) within the framework of this research project’*.

### 3.4 Let's Discuss Health users’ experience and its impact on the visit

In view of the questionnaire data, patients and HCP were generally satisfied with LDH and that LDH contributed to improved communication during the medical encounter (Table 4). Both patients and HCP indicated high agreement with the following statements: priority setting (HCP: 87%); question asking (Pt: 94%); checking understanding (Pt:88%); and expressing concerns early in the visit (HCP: 89%).

Once again, patients and HCP expressed divergent opinions during focus group discussions. Most patients considered that LDH helped them structure and prioritize their reasons for consultation, providing more complete and accurate information thus achieving their goals for their medical visit.

*‘When we prepare well it allows the doctor to know us better since we give more information. It makes the doctor happy. It allows you to prepare for the real questions that the doctor will ask. It helps the MD to understand all the necessary follow‐ups, to see that we are doing our homework well’*.

*‘When we prepare well, we know what we have to ask, we describe our symptoms well in advance, we think of something we write it down immediately, our appointments get better. We make our doctor happy to see that we did our job’*.

Some HCP perceived a sense of pride in their patients, hypothesizing it is because they had been able to share all the information that was important to them and help them achieve their goals.

*‘He (the patient) said to me, “ I thought of issues that I would have forgotten without… (LDH)”’*

On the other hand, HCP found that preparing with LDH sometimes leads patients to ask unnecessary or irrelevant questions such as illustrated in this HCP citation.

*‘You know it wasn’t a big deal, but there was one who wanted to know when she would pass her EKG’*.

### 3.5 Barriers to the implementation of Let's Discuss Health (LDH)

Table 5 reports the main barriers to LDH use from both patients and HCP perspectives based on data from the project coordinators’ logbooks and focus group discussions. Reported barriers vary by type of participants. In general, patients’ views related to their interest in using LDH, while the HCP’s data highlighted the difficulties in changing their clinical routines.

**Table 5:** Barriers to implementation of Let's Discuss Health (LDH) website

PATIENTS’ OPINIONS	HCP’ OPINIONS
• Lack of interest. • Limited access to technology: do not have computer or Internet. • Difficulty or uneasiness navigating the Internet or technical issues with the website. • Time constraints: time consuming to visit website, explore the tools and complete all questions. • Language barriers: problematic for patients who, although they speak French, do not read or write it.	• Low patient literacy level is problematic and an important limiting factor to LDH use in some of the clinics. • Time management issues. ‐ Fear of longer consultations due to a potentially longer list of issues and concerns the LDH prepared patients bring to the consultation. ‐ Fear of loss of control of unfolding of consultation.

Moreover, the results from the semi‐structured interview with project coordinators highlighted the following difficulties: (a) mobilizing staff while major reforms were occurring in Quebec's Health and Social Services that resulted in staff shortages; (b) managing research documents (patient consent forms, follow‐up with patients because study questionnaires were poorly completed, work involved in identifying eligible patients from HCP‐patient panels in the electronic medical record (EMR), etc) and (c) insufficient budget for the project resulting in significant in‐kind contributions from the clinic that had not been anticipated.

### 3.6 Suggestions to improve Let's Discuss Health (LDH) implementation in primary care clinics

Study participants (patients and HCP) offered the following suggestions to improve LDH implementation: (a) technical support to showcase the LDH website: bookmarks, posters, telephone and e‐mail reminders, HCP personally inviting patients to visit the LDH website, a video demonstrating functionalities on the clinic waiting room TV screens; (b) human resources: onsite volunteers introducing patients to LDH website in the waiting room to support implementation; (c) partnership building: HCP, patients and clinic managers need to work together to facilitate the practice changes, both administrative and clinical, involved with the implementation of LDH in the clinic's workflow.

## 4 DISCUSSION

The aim of this research was to assess the potential for integration of LDH in routine PC clinical visits of chronically ill patients, as well as to identify the barriers and benefits of adoption of LDH. Overall, 16.5% of invited patients visited the website and completed all questionnaires. Although this proportion is apparently low, it is comparable to what is generally found in the literature on PHR uptake.^45^, ^46^ Krist et al (2012)^34^ reported that 16.8% of patients had used a web portal made available to them after a mailed invitation. However, this same group of researchers found that after providing practices basic implementation assistance that included clinic PHR champions, HCP and staff training, patient PHR adoption rate reached 25.6%.^47^ The practices in our study that involved medical students and residents in the implementation process thus provided a form of ‘implementation assistance’ probably accounting for the increased uptake of LDH by patients in two of the practices. As expected, fewer patients with lower education status responded to our invitation to prepare for their encounters using LDH, a form of health IT.^46^ Poor computer skills, lack of Internet access, low health literacy and limited physical and cognitive abilities all contribute to what has been coined the ‘digital divide’.^48^ These findings echoed the HCP's concerns regarding the exclusion of people with low literacy skills, because these persons are also less likely to use health information technology tools. The potential exclusion of people who are more vulnerable, such as the elderly and those with low health literacy, is often evoked by HCP as a reason to oppose **the** implementation of innovations in e‐Health. If it is true that consulting online information is generally associated with higher levels of education, however, recent surveys show that disparities in access to the Internet are diminishing when the growing popularity of mobile devices **is** taken into account, including the elderly.^49^, ^50^ Thus, there are fewer reasons to eschew the use of online tools to activate patients. Indeed, in the United Kingdom, the National Health Service (NHS) recommends doctors prescribe these applications because they can strengthen doctor‐patient communication.^47^

This study presents several benefits for the participants. LDH has bridged a gap in terms of the availability of French language technological tools that engage patients in self‐care and support collaboration with their HCP. It provided patients with communication tools which proved acceptable and transferable into routine clinical practices. Most patients indicated a favourable evaluation of the website: its functionalities and their usefulness in helping them get their *voice heard*. In general, patients reported a positive impact on the use of the website in that it helped them to: (a) adopt an active role in the health‐care encounters with their HCP; (b) define and recall their visit agenda and reduce health‐care encounter‐related stress; (c) feel more confident to ask questions; (d) feel more motivated to prepare their future medical visits; and (e) improve their chronic illness management. Patient focus group discussions confirmed these findings which are very similar to reported studies on agenda setting.^32^ HCP reported that patients presented with clear reasons to consult and that the LDH visit preparation summary was easy to integrate into routine visits. However, a certain disconnect emerged between HCP and patient perceptions as to the value of LDH in promoting a sense of partnership and collaboration. Some HCP did not feel that the visit preparation summary provided new information or that it helped organize the visit. It was as if their expectations of the role of the summary were not met. A few HCP commented for example that the visit preparation summary did not provide information on important ‘red flags’ in relation to reported symptoms. This could explain the absence of ‘perceived added value’, of the visit summary expressed in the HCP focus groups. In this regard, we would like to point out that the summary sheet was conceptualized not as a ‘mini’ case history useful for the HCP but as a tool to help the patient clarify their own agenda for the encounter and organize the information, he/she wished to share with their HCP. Moreover, the concept of ‘agenda setting’ at the beginning of any HCP‐patient encounter is recognized as the keystone of an effective medical encounter and well‐aligned consultations.^51^, ^52^ This is true for initial and follow‐up visits in the long‐term management of these patients as recognized in Wagner's Chronic Care Model.^52^, ^53^, ^54^, ^55^ The LDH was created to help patients prepare, organize and prioritize topics for discussion for their upcoming visit thus enabling them to engage in a collaborative visit agenda setting with their HCP.^56^, ^57^ These aspects do not seem to have been considered important in the opinions expressed by the HCP.

During the focus group discussions, HCP were divided with regard to the impact of **patients** use of LDH on the encounter length. According to some HCP, LDH seemed to lengthen encounters, while others reported that they did not note any changes. A previous study which evaluated the efficacy of web‐based educational approaches on patient‐provider communication did not find any change of the encounter length.^15^ Some HCP expressed some doubts about LDH’s usefulness and their reluctance to integrate it in their practice because of their concern of being overwhelmed by questions and needing to devote more time to appointments. Because of this, some of them were uncertain of their intention to continue to encourage LDH use. In their view, encouraging patients to use LDH required an additional effort on their part which they were not necessarily ready to commit to. Similar to our findings, many studies have shown that HCP express many concerns about use of patient portals.^58^, ^59^ However, Wittink et al (2018) showed that using a health information technology (HIT) designed to help complex patients with multimorbidity disclose their stressors to their HCP was associated with increased psychosocial concerns and stressors disclosure without increasing visit length.^60^ These disclosures also arrived earlier in the encounter. Yet, successful adoption of patient portals and visit preparation web‐based tools depends on their integration into regular care programs and the recognition that active patient participation is an integral part of effective patient‐HCP relationships.^45^, ^58^

Patients who participated in the study were highly activated as indicated by high PAM scores (93% of patients had 3 or 4 level PAM score), which is consistent with Hibbard and Green's study^61^ as well as Henselmans’ study.^62^ However, in the absence of a patient baseline PAM score, it is not possible to attribute the level of activation observed to the LDH implementation. Our research design, a real‐world implementation study, did not allow us to evaluate a change in patient PAM scores before and after the use of LDH as questionnaires were completed by patients after they had experienced the use of LDH. It is unlikely that the use of LDH could have had a huge impact on the activation score considering the elements that are measured in this instrument. However, it makes sense that already activated patients could have been more attracted to the type of self‐management support LDH offered than less activated patients.

Patients view LDH as an opportunity to take responsibility for their own health‐care and play a greater role in it. Its use contributes to building a better understanding of their health problems. Both data from questionnaires and focus groups showed that patients intended to continue using LDH and even, for some of them, encourage their relatives and acquaintances to use it. This is interesting as it is a robust indication of the perceived usefulness of LDH as a self‐management tool. Divergent pictures of HCP perspectives about the perceived usefulness and impact of LDH emerged from the two sources of data collected. Post‐visit questionnaire responses by HCP generally indicated quite a favourable evaluation of patients’ use of LDH. However, during focus group discussions, although they maintained their initial positive evaluation of patient behaviours, HCP explained that the question format of the post‐visit questionnaire did not allow them to express if they noted a ‘change’ from their patients’ usual behaviour. HCP attributed the lack of change to the fact that patients who accepted to participate were those that already prepared well and were well organized. The high PAM scores noted in study participants seem to confirm HCP’s impressions.

The barriers to the use of LDH identified by patients are identical to those found in the literature.^62^, ^63^, ^64^, ^65^, ^66^, ^67^ They include lack of interest, limited access to technology (no computers or no Internet), limited Internet knowledge or not comfortable with the Internet, lack of time and language barriers. The adoption of LDH by the HCP and its integration into routine clinical visits remains fragile, and several obstacles are still present. Among other things, HCP identify the low level of patient literacy as a barrier as well as their own lack of time to make the necessary changes that would be required in their clinical practices workflows such as systematically inviting patients to prepare their visits, making sure they access and manage the patient's visit preparation summary in a timely manner and integrating the patient's priorities into the visit agenda setting. As stated by Archer et al^68^ there is a need to better **teach** HCP on how these tools may support patient engagement, and self‐care and to downplay the problems they anticipate due to use of such tools by their patients.

Many ideas to facilitate the use of the LDH were suggested by study participants. Among others, they suggested having a video demonstrating LDH functionalities projected in clinic waiting rooms, implementing automated reminders to visit LDH before the scheduled appointment, including a hyperlink to LDH website in the clinic's own websites, allowing patients to electronically share their visit preparation summary with the HCP before the scheduled visit. However, unlike some patient health records (PHR) used in Anglo‐Saxon countries,^33^ LDH currently does not offer any capacity to interface with the clinic's electronic medical record (EMR). Such a secure bidirectional interface would allow patients to access health information from their medical records and insert it directly into LDH and allow HCP to access in a timely fashion the contents of the patient's visit preparation summary. It was believed necessary that the various stakeholders will need to intervene in a coordinated manner to promote and facilitate the use of LDH and that a simple invitation to visit LDH will never be sufficient to promote its use in a meaningful way.

Of interest, HCP did not mention the role they could play to encourage LDH use by their patients, possibly because they did not recognize its added value for patients. Getting patients to prepare for the medical encounter is probably one of the most powerful ways to address the inherent asymmetry of the doctor‐patient encounter.^69^, ^70^, ^71^, ^72^ Thus, not perceiving the importance of the role they play in encouraging patient participation represents a significant barrier to the adoption of LDH. Patients value their trusted HCP’s opinion and would probably consider this suggestion to prepare as an explicit invitation to partner with them. For example, HCP checking explicitly if the patient has prepared for the visit using LDH during the encounter could go much further than a letter or phone call.

### 4.1 Study limitations

This study has several limitations. First, because we did not reach the planned sample size, of 150 patients per province, we could not compare the data according to the two provinces (Quebec vs New Brunswick) to identify differences, especially in regard to the perceived value of LDH, and its perceived impact on both patient participation in the visit as well as its impact on its organization. Second, since we did not audio record the encounters, we do not know how often or to what extent the patients and HCP referred to the patient summary sheet and how it influenced, for example, visit priority agenda setting. Lastly, this study is subjected to a patient selection bias as discussed in the previous section; it is possible that patients who accepted to participate were already highly activated patients.^73^In this self‐selected patient group, however, the usefulness of LDH was confirmed.

## 5 CONCLUSION

LDH has fulfilled its promise for patients that have used it but the picture is less clear for the HCP. The findings of our study contribute in filling a knowledge gap on how to implement the use of electronic tools which assist patients in the preparation of their medical visits. Our study suggests a strong need for training and support to assist less activated patients with portal registration and use, particularly those with limited health literacy. Efforts to encourage electronic tool use among these patients should directly address health literacy and support access for caregivers. Choosing to use LDH moves patients from a ‘passive’ stance in the medical encounter to an ‘active’ one. This change of behaviour is important and as any change of behaviour it will not happen overnight. It will take several requests from staff and HCP before we observe the transition from a passive to an active attitude. This attitude change is likely to help the HCP to organize visit priorities and to increase the success of the proposed treatments.

The study needs to be replicated in other medical environments using larger and more diverse samples. In future studies, it would be relevant to analyse communication patterns during encounters prepared with LDH. In this context, it would be interesting, for example, to measure results on visit agenda setting, patient initiated discussions, amount of dialogue^15^ and patient recall and adherence to the recommendations and interventions discussed during the encounter.

In terms of next steps in our own context, we are exploring the possibility to include LDH into the Québec Health Booklet, because of the complementary functionalities it provides to those already present in this provincial web platform such as access to laboratory test results, imaging reports and making medical appointments. The Québec Health Booklet would thus become even more **patient‐centred** than it is at the moment.

## CONFLICT OF INTEREST

Both BD and CR, as project coodinators, received honoraria for their work in this project. MTL and CR are married. The authors have no other potential conflict of interest or financial relationship relevant to this article to disclose.

## AUTHORS’ CONTRIBUTION

MTL was the principal investigator for the study. She wrote the protocol and supervised the protocol submission to all funding agencies and ethics review boards. She supervised the conduct of the study in all sites. MTL and CR conducted the qualitative interviews in one of the two provinces. She developed the analysis plan and supervised all quantitative and qualitative analyses. She planned the first draft of the article and supervised Ms Diallo, the study coordinator, at every step of the draft. She reviewed and contributed significantly to all versions of the paper. CR supervised the qualitative analyses and participated in the interpretation of all the data. He contributed to the original draft and commented on all revisions. BD analysed the data, both quantitative and qualitative, with input from all authors. Under MTL’s supervision, she wrote the first draft of the paper and integrated the revisions from all the authors. NB and JJ were responsible for the study conduct in one of the provinces, and NB conducted the qualitative interviews in that province. NB, JJ, EB and HW read the draft and approved the final version.

## Data Availability

The data that support the findings of this study are available from the corresponding author upon reasonable request.
